# Mitochondrial maintenance as a novel target for treating steroid-induced osteonecrosis of femoral head: a narrative review

**DOI:** 10.1530/EOR-24-0023

**Published:** 2024-11-08

**Authors:** Yidan Yang, Yi Jian, Youwen Liu, Maoxiao Ma, Jiayi Guo, Bin Xu, Chen Yue

**Affiliations:** 1 Henan University of Chinese Medicine, Zhengzhou, Henan Province, People’s Republic of China; 2 Department of Orthopedics, Luoyang Orthopedic Hospital of Henan Province, Orthopedic Hospital of Henan Province, Luoyang, Henan Province, People’s Republic of China; 3 Department of Orthopedics, Tongde Hospital of Zhejiang Province, Hangzhou, Zhejiang Province, People’s Republic of China

**Keywords:** mitochondria dynamics, mitochondrial homeostasis, mitophagy, steroid-induced osteonecrosis of the femoral head

## Abstract

- The pathogenesis of steroid-induced osteonecrosis of the femoral head (SONFH) remains unclear; however, emerging evidence suggests that mitochondrial injury plays a significant role.
- This review aims to elucidate the involvement of mitochondrial dysfunction in SONFH and explore potential therapeutic targets.
- A comprehensive literature search was conducted in PubMed, Web of Science, and Elsevier ScienceDirect, focusing on mitochondrial homeostasis, including mitophagy, mitochondrial biogenesis, mitochondrial dynamics, and oxidative stress in SONFH. Ultimately, we included and analyzed a total of 16 studies.
- Glucocorticoids initially promote but later inhibit mitochondrial biogenesis in osteoblasts, leading to excessive ROS production and mitochondrial dysfunction. This dysfunction impairs osteoblast survival and bone formation, contributing to SONFH progression.
- Key proteins such as mitochondrial transcription factor A (TFAM) and peroxisome proliferator-activated receptor γ coactivator 1-α (PGC1α) are potential therapeutic targets for promoting mitochondrial biogenesis and reducing ROS-induced damage.
- Enhancing mitochondrial function and reducing oxidative stress in osteoblasts may prevent or slow the progression of SONFH. Future research should focus on developing these strategies.

## Introduction

**Figure 1:**
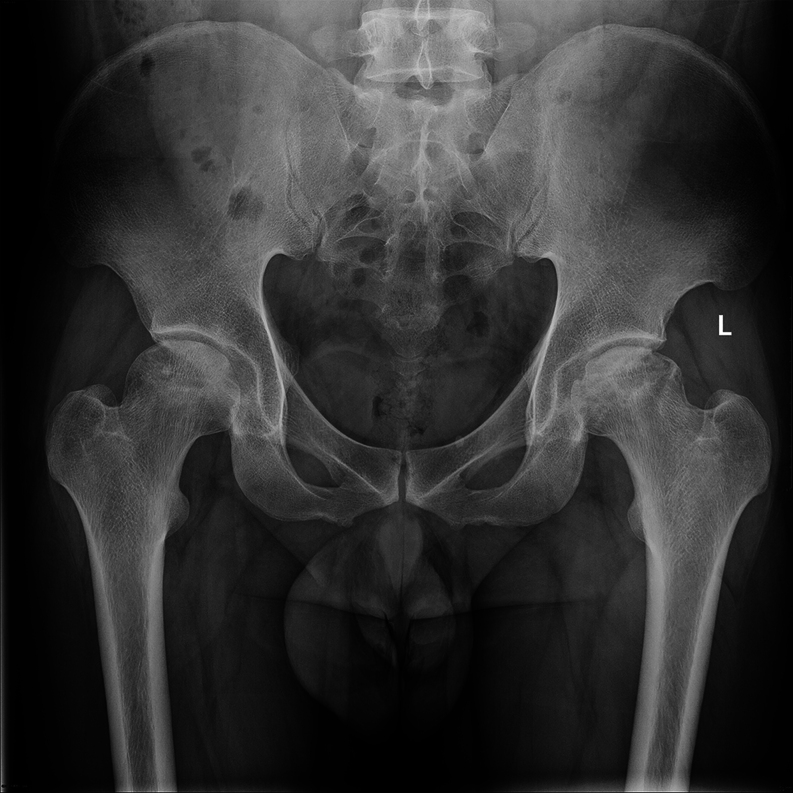
Imaging Characteristics of SONFH Patients. The imaging photograph depicts a male patient suffering from bilateral steroid-induced osteonecrosis of the femoral head. The right femoral head is at the pre-collapse stage, classified as ARCO II, while the left femoral head has already collapsed, classified as ARCO III.

Steroids, widely used to treat various inflammatory and autoimmune diseases (1), can increase the risk of femoral head necrosis with long-term or high-dose use (2), which in severe cases can lead to disability (3). Steroid-induced osteonecrosis of the femoral head (SONFH) may account for 26.35% of all cases of femoral head osteonecrosis among men and 55.75% of cases among women (4). However, due to a lack of effective early intervention in clinical settings, over 80% of SONFH patients require total hip arthroplasty (5). The imaging results for patients with SONFH are presented in Fig. 1.

While the pathogenesis of SONFH is still entirely unclear, steroids are thought to injure vascular endothelial cells, perturb osteogenic differentiation, and induce hyperactivity of osteoclasts as well as apoptosis of osteoblasts, osteocytes, and bone marrow mesenchymal stromal cells (BMSCs) (6, 7). These alterations interfere with bone remodeling, leading to the local loss of bone mass and potentially causing femoral head collapse (7, 8). At present, drugs such as bisphosphonates and calcitonin are used to treat early stages of SONFH. Although these drugs can inhibit osteoclast activity and show good clinical effects in the early stages of SONFH, they cannot sustain effective bone turnover (9).

Glucocorticoids (GCs) significantly impact bone cell proliferation, differentiation, function, and apoptosis. They inhibit osteoblast differentiation by promoting adipogenic factors and suppressing the Wnt signaling pathway, reduce osteoblast proliferation, increase oxidative stress, and elevate reactive oxygen species (ROS) production. Prolonged exposure leads to mitochondrial dysfunction and apoptosis of osteoblasts and osteocytes by disrupting mitochondrial membrane potential and releasing pro-apoptotic factors like cytochrome C. These effects contribute to bone loss and osteoporosis (10).

Emerging evidence suggests that SONFH involves mitochondrial injury (11), making it a potential novel therapeutic target. Mitochondria are essential for cellular energy production, regulating oxidative stress, and apoptosis. Steroids can disrupt mitochondrial homeostasis, leading to excessive ROS production and apoptosis. Impaired mitochondrial function due to steroid exposure can exacerbate tissue damage and necrosis. Thus, understanding and targeting mitochondrial dysfunction could provide new avenues for treating SONFH. Disruption of mitochondrial homeostasis has already been implicated in other diseases (12, 13), and it is known to impair osteogenic differentiation (14, 15). The steroid dexamethasone appears to induce apoptosis in osteoblastic cells through a pathway mediated by mitochondria (16). Bone cell apoptosis in SONFH appears to involve the impairment of mitochondrial function and the release of cytochrome C (17).

Based on relevant research published in recent years, we carried out this review to expound on the relationship between mitochondrial homeostasis and the pathogenesis of SONFH, including the potential mechanisms of mitophagy, mitochondrial biogenesis, mitochondrial dynamics, and ROS generation, with the aim of assisting orthopedists in gaining a comprehensive understanding of these mechanisms and providing a novel target for SONFH treatment.

## Searching strategies

This narrative review is conducted and reported according to the Scale for the Assessment of Narrative Review Articles (SANRA) guidelines to ensure a structured and comprehensive review process (18).

We searched the literature in PubMed, Web of Science, and Elsevier ScienceDirect. The search strategy was as follows: (osteonecrosis of the femoral head or aseptic necrosis of the femoral head or steroid-induced osteonecrosis of the femoral head or glucocorticoid-induced osteonecrosis of the femoral head or ONFH or ANFH or SONFH or GONFH) and (mitochondrion or mitochondrial dysfunction or mitochondrial homeostasis or mitophagy or mitochondrial biogenesis or mitochondrial dynamics or oxidative stress).

The research explored the role of mitochondrial homeostasis (mitophagy, mitochondrial biogenesis, mitochondrial dynamics, and mitochondrial ROS metabolism) in SONFH. The exclusion criteria are as follows: (i) Studies that did not specifically explore mitochondrial homeostasis, including mitophagy, mitochondrial biogenesis, mitochondrial dynamics, or mitochondrial ROS metabolism in the context of SONFH, were excluded; (ii) Research that did not involve subjects relevant to SONFH, such as studies on other types of osteonecrosis or unrelated diseases, was excluded; (iii) Review articles, case reports, and studies without experimental or clinical data related to mitochondrial functions in SONFH were excluded; (iv) Studies that lacked sufficient data or had inconclusive results regarding the role of mitochondria in SONFH were excluded.

Literature selection followed these steps: first, all retrieved studies were imported into Endnote X7 and duplicates were excluded. Then, irrelevant literature was excluded by two independent researchers based on titles and abstracts. Finally, the same two researchers included the studies that met the selection criteria after scrutinizing full texts. Any disagreement was resolved by discussion with a third researcher.

Following the aforementioned retrieval strategy, a total of 262 studies were identified through a search. After removing duplicates, 108 studies were left, and then 75 studies were excluded based on the irrelevance of their titles and abstracts. Additionally, 17 studies were removed because they did not meet the selection criteria. Finally, 16 studies were included in this review.

## Mitochondrial homeostasis

**Figure 2:**
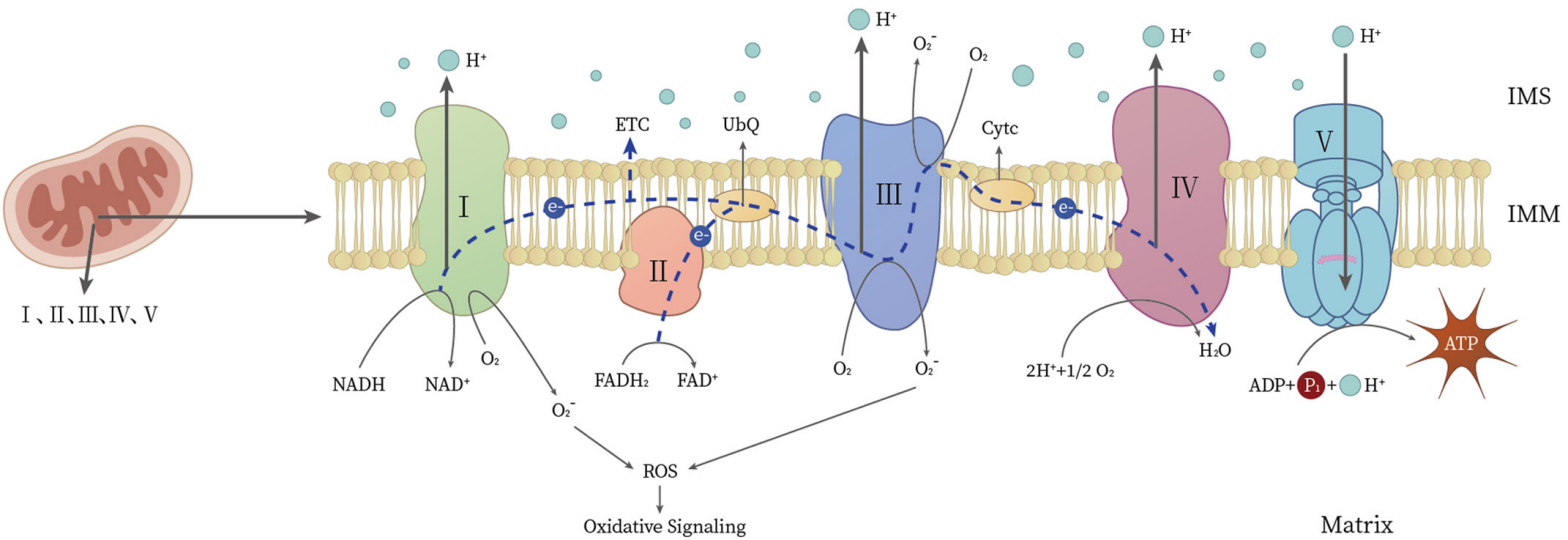
The physiological process of mitochondrial ATP and ROS generation. Mitochondria utilize a series of enzymes and complexes in the ETC to generate ATP through oxidative phosphorylation. During this process, electrons are transferred along the ETC, leading to the pumping of protons across the inner mitochondrial membrane. This establishes an electrochemical gradient, which drives ATP synthesis by ATP synthase (V). Simultaneously, a small fraction of electrons leaking from the ETC can react with molecular oxygen, resulting in the formation of ROS. I, NADH Reductase; II, Succinate Dehydrogenase; III, Cytochrome Reductase; IV, Cytochrome Oxidase; ETC, electron transport chain; UbQ, ubiquinone; Cytc, cytochrome c; IMS, mitochondrial intermembrane space; IMM, inner mitochondrial membrane.

The mitochondrion acts as a hub of metabolic and signaling processes. It comprises four functional regions: outer membrane, intermembrane space, inner membrane, and matrix. These four regions participate in oxidative phosphorylation to produce ATP, fatty acid oxidation, calcium buffering, phospholipid synthesis, generation of ROS, maintenance, synthesis of iron-sulfur clusters, and innate immune signaling (19). Mitochondria are crucial for energy production through oxidative phosphorylation, which occurs in the inner mitochondrial membrane. The electron transport chain (ETC) drives the synthesis of ATP by creating a proton gradient used by ATP synthase. During this process, ROS are produced as by-products. While ROS are vital for cell signaling, excessive ROS can cause oxidative stress, leading to cellular damage. Maintaining mitochondrial homeostasis, therefore, involves a balance between ATP production and the management of ROS levels (Fig. 2).

The cell’s population and morphology of mitochondria are highly dynamic (20). The lifespan of mitochondria is short, and the function of new mitochondria noticeably declines after only a few days. This means that mitochondria are essentially in an environment of continuous exposure to high levels of ROS (21, 22), which damage mitochondrial proteins and induce mutations in mitochondrial DNA (23). Older mitochondria continuously undergo fission and fusion to produce new mitochondria, or they undergo mitophagy to be removed from the cell as waste (24). During their function, mitochondria must maintain a robust antioxidant system that can keep levels of potentially toxic ROS in check. The balance among mitochondrial biogenesis, mitophagy, and the dynamics of mitochondrial fusion/fission – collectively referred to as ‘mitochondrial homeostasis’ – is important for maintaining a stable pool of functional mitochondria in the cell (25).

When damaged mitochondria cannot be degraded by mitophagy, the normal ROS metabolism becomes disordered, and abundant Ca^2+^ and cytochrome C are released into the cytosol, triggering cell apoptosis. In this way, mitochondrial damage has already been implicated in diabetes, neurodegenerative diseases, and the effects of aging (12, 13).

## Mitochondrial homeostasis in SONFH

While the pathogenesis of SONFH remains unclear, the most widely accepted mechanism involves apoptosis of osteogenic cells, abnormal osteogenic differentiation, and impaired microcirculation (6). Recently, a study demonstrated that glucocorticoids may inhibit the transcriptional activity of glucose transporter member 1 (GLUT1) in SONFH, which is responsible for transporting glucose from the extracellular space into the cell. This inhibition reduces the amount and activity of GLUT1 in cells, leading to decreased mitochondrial activity, reduced ATP production, the promotion of apoptosis, and inhibition of osteoblast ossification via the GC/GR/GLUT1 axis (26). In addition, another study found that BID and FTH1, two genes involved in the mitochondrial apoptotic pathway, were highly expressed in SONFH and promoted apoptosis of bone cells (27).

A key driver of the disease appears to be oxidative stress: long-term and/or high doses of corticosteroids within the hypoxic environment of the femoral head produce more ROS than osteogenic cells can remove (17, 28, 29). These steroids can increase mitochondrial activity, leading to higher levels of oxidative phosphorylation, but they cause a decrease in the activity of antioxidant enzymes such as SOD1, HO-1, and catalase, which in turn generates more ROS (30). The abundant ROS disrupts the outer and inner mitochondrial membranes, disturbing oxidative phosphorylation, resulting in a decrease in energy production. Furthermore, the damaged membranes allow the leakage of Ca^2+^ and cytochrome C into the cytosol, the fluid within the cell. Mitochondrial dysfunction occurs when cytochrome C, crucial for ATP production, is released into the cytoplasm, reducing ATP and affecting energy supply; this release also triggers apoptosis by forming a complex with apoptotic protease activating factor-1 and caspase-9, activating the caspase cascade. Excess Ca^2+^ can lower mitochondrial membrane potential, decrease ATP synthesis, increase membrane permeability, and release pro-apoptotic factors, while both cytochrome C release and Ca^2+^ influx elevate oxidative stress, leading to cell damage and apoptosis (29, 31, 32). This inhibits osteogenic differentiation and bone mineralization function (33, 34, 35).

Mitochondria injury also induces the apoptosis of bone microvascular endothelial cells (36), reducing blood flow to the femoral head and creating a hypercoagulable state conducive to ischemia and thrombus formation (37). Ischemia and hypoxia of the femoral head impair the function of the oxygenated mitochondrial respiratory chain to produce excessive ROS, exacerbating the development of SONFH. Oxygen is crucial for the operation of the mitochondrial ETC. Under ischemic and hypoxic conditions, mitochondria are unable to carry out normal electron transfer, leading to the accumulation of electrons between various complexes of the ETC. Electrons are prone to leak from ETC complexes I and III into the mitochondrial matrix, reacting with oxygen molecules to generate superoxide (O_2_^−^), a type of ROS, which leads to the overexpression of SOD2. SOD2 is an upregulated gene in SONFH, and this may lead to the accumulation of hydrogen peroxide (27). An excessive increase in oxides leads to mitochondrial dysfunction and ultimately cell apoptosis.

P53, a tumor suppressor protein, contributes to the apoptosis of osteogenic cells in the femoral head. After being activated by the glucocorticoid receptor, p53 translocates to the mitochondria, where it forms a complex with cyclophilin-D (38), disrupting the normal mitochondrial membrane potential. Additionally, oxidative stress can upregulate p53 in BMSCs, further exacerbating mitochondrial dysfunction (33).

Ferroptosis, a distinct form of programmed cell death characterized by mitochondrial damage, can lead to cell death via iron-dependent mechanisms. It has been shown to play a role in the pathogenesis of femoral head necrosis. A recent study has shown that increased p53 expression inhibits the expression of SLC7A1 and GPX4 in BMSCs, MC3T3-E1, and MLOY4 cells in SONFH, leading to a reduction in intracellular GSH levels. This reduction results in elevated levels of MDA, ROS, and lipid ROS within the cells, causing mitochondrial shrinkage, a decrease in mitochondrial cristae, and impaired mitochondrial function, ultimately leading to ferroptosis and abnormal differentiation of osteoblasts and apoptosis of bone cells (39, 40). FTH1 gene associated with ferroptosis is highly expressed in SONFH (27). Antioxidants such as melatonin (MT) have been shown to attenuate GCs-induced ROS generation and promote osteogenic differentiation by inhibiting ferroptosis mediated through MT2, which can relieve the SONFH process (40).

Furthermore, mitochondrial dysfunction may affect the differentiation of adipocytes, promote apoptosis of bone cells, and accelerate the process of SONFH. The Wnt/β-catenin pathway is crucial for regulating the differentiation of BMSCs, with increased expression promoting osteoblast development (41, 42). In SONFH rats, glucocorticoid exposure has been shown to disrupt the osteogenic and adipogenic differentiation of BMSCs by inhibiting β-catenin signaling (43). In another study, it was also proven that adipocyte formation was promoted and bone cell apoptosis was accelerated when the Wnt/β-catenin pathway was inhibited in SONFH (42). Additionally, mitochondrial damage produces excessive ROS, which promote the binding of FoxO to β-catenin, ultimately leading to reduced osteoblast formation (41).

These considerations suggest that mitochondrial dysfunction is associated with the development of SONFH, and restoring the function of mitochondria may be an effective treatment against SONFH. The following sections elaborate on the pathophysiological mechanisms and the potential treatment of molecular targets involved in the four important components of mitochondrial homeostasis: mitophagy, mitochondrial biogenesis, mitochondrial dynamics, and mitochondrial ROS metabolism.

## Mitophagy

**Figure 3:**
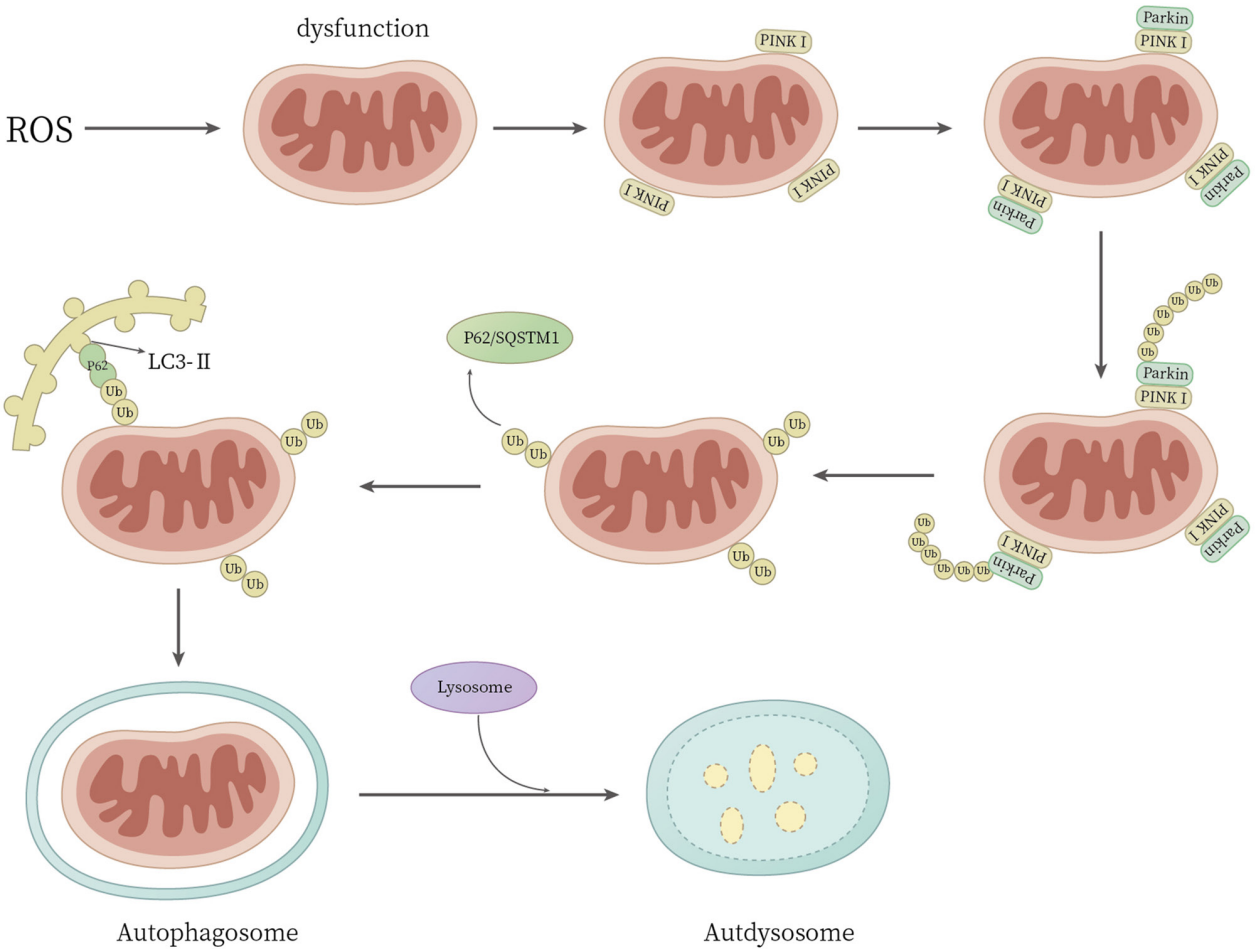
Process of Mitochondrial Autophagy. When mitochondrial inner membrane depolarization occurs due to oxidative stress, the Pink1/Parkin system serves as a sensor for mitochondrial quality and is activated. PINK1 accumulates on the outer mitochondrial membrane, phosphorylating ubiquitin and recruiting the E3 ubiquitin ligase Parkin from the cytosol to the outer membrane. Parkin then ubiquitinates several proteins in the outer membrane, marking the damaged mitochondria for degradation. Simultaneously, specific receptors on the mitochondrial outer membrane identify damaged mitochondria, recruiting autophagy-related proteins to initiate the formation of autophagosomes around the damaged organelles. Adaptor proteins such as p62/SQSTM1 bind to ubiquitin moieties on the outer membrane and autophagic protein LC3-II on autophagosomes, facilitating the sequestration of damaged mitochondria within autophagosomal membranes. The autophagosome subsequently fuses with lysosomes, forming autolysosomes, where the damaged mitochondria are degraded by lysosomal enzymes.

Mitophagy, in which damaged or ineffective mitochondria are delivered to autophagosomes for degradation (44), is one of the major mitochondrial quality control processes (45). An appropriate level of mitophagy is required to prevent oxidative stress and apoptosis by reducing ROS and proapoptotic factors released by the damaged mitochondria (46). Oxidative stress initially promotes mitophagy in BMSCs via a pathway involving c-Jun N-terminal kinases, but later it inhibits mitophagy and promotes apoptosis (47). When oxidative stress damages mitochondria, leading to depolarization of their inner membranes, the Pink1/Parkin system acts as a sensor for mitochondrial quality and is activated (48). PINK1 and Parkin function as the first steps of a signaling pathway that activates mitochondrial quality control pathways in response to mitochondrial damage. PINK1 accumulates on the outer mitochondrial membrane, where it phosphorylates ubiquitin and recruits the E3 ubiquitin ligase Parkin from the cytosol to the outer membrane. Parkin ubiquitinates several proteins in the outer membrane, and the adaptor proteins p62/SQSTM1 bind to these ubiquitin moieties and autophagic protein LC3-II on autophagosomes to mediate sequestration of the organelle in an autophagosomal membrane, allowing mitophagy to proceed (48, 49, 50) (Fig. 3).

Deficiency of PINK1 inhibits osteoblast differentiation and increases mitochondrial production of ROS (15). Upregulating Parkin exerts analogous effects, but only if P53 is simultaneously downregulated (33), probably because P53 can inhibit the activity of Parkin by binding to its RING0 region (46, 51), and downregulation of P53 can effectively reduce the decline of mitochondrial membrane potential (38). Dexamethasone appears to inhibit hypoxia-induced mitophagy by downregulating BNIP3, NIX, and LC3-II, which are critical for the mitophagy process. BNIP3 and NIX are pro-apoptotic proteins that promote the removal of damaged mitochondria by mediating their recognition and engulfment by autophagosomes, while LC3-II is essential for the formation and elongation of autophagosomes. This inhibition, therefore, promotes apoptosis of osteocytes. These effects of dexamethasone can be reversed by overexpression of Hypoxia-inducible factor-1α (52). A study has shown that high-dose hormones can effectively inhibit the expression of PINK1, Parkin, and LC3-II, upregulate p62, significantly weaken the autophagy function of osteoblast mitochondria, and affect the survival rate of osteoblasts (53). However, Vitamin K_2_ administration can significantly attenuate the dexamethasone-induced downregulation of LC3-II, PINK1, and Parkin in osteoblasts, thereby restoring mitophagic processes and normal osteoblastic activity (53).

## Mitochondrial biogenesis

While undifferentiated BMSCs and osteoblasts show relatively weak mitochondrial activity, osteogenic induction induces a large increase in oxygen consumption. To provide this additional metabolic power, mitochondrial biogenesis is strongly upregulated (35, 54). Glucocorticoid initially promotes mitochondrial biogenesis in osteoblasts but later inhibits it (55). The resulting excess levels of ROS may further inhibit biogenesis by interfering with the cytosolic synthesis of mitochondrial proteins and their targeting to the appropriate submitochondrial compartments (25, 56).

Several proteins promote osteogenesis by promoting mitochondrial biogenesis. For example, adding mitochondrial transcription factor A (TFAM) to osteocytes under oxidative stress protects them from apoptosis (57). Taurine can reverse the downregulation of TFAM in osteocytes under glucocorticoid and hypoxia stimulation and prevent SONFH in rabbits (58). The protein ‘peroxisome proliferator-activated receptor γ coactivator 1-α’ (PGC-1α) interacts with the transcription factors NRF1 and NRF2, which in turn stimulate the expression of TFAM to promote mitochondrial biogenesis (59), and the polypeptide hormone liraglutide, which is a glucagon-like peptide-1 (GLP-1) receptor agonist, stimulates signaling involving the GLP-1 receptor, cyclic AMP, phosphorylated AMP kinase, and the Adipo1 receptor to promote mitochondrial biogenesis and osteogenesis (60). Resveratrol, a polyphenolic compound found in various plants, can activate sirtuin 1 and rescue mitochondrial biogenesis in osteoblasts that have been exposed to dexamethasone (61). A neuroprotective bovine colostrum has been shown to attenuate mitochondria-induced apoptosis in osteoblasts treated with dexamethasone, potentially by activating the Hsp70 system to correct and stabilize the structure of proteins necessary for mitochondrial biogenesis (62, 63). These examples build a strong case that these proteins could be promising targets. Regulating their activity may enhance mitochondrial biosynthesis in osteoblasts, thereby promoting osteogenesis and aiding in the repair of femoral head necrosis.

## Mitochondria dynamics

New mitochondria can be generated from old ones through fusion mediated by mitochondrial fusion protein and optic atrophy protein 1 (64), and by fission mediated by dynamic-associated protein 1 (DRP1), mitochondrial fission protein 1 (FIS1), and mitochondrial fission factor (MFF) (23). These two processes must be kept within appropriate limits as part of mitochondrial homeostasis. Excessive fission, for example, can lead to abnormal morphology, permeabilization of the outer mitochondrial membrane, release of ROS, and cell death (65). As glucocorticoids increase mitochondrial fusion, it initially enhances mitochondrial function. However, when followed by excessive fission, it leads to mitochondrial dysfunction by promoting the release of pro-apoptotic factors like cytochrome C into the cytosol, ultimately resulting in the apoptosis of osteoblasts (55).

Treating endothelial progenitor cells with glucocorticoid methylprednisolone increases the number of granulated mitochondria, raises the level of ROS, upregulates MFF and FIS1, and eliminates normal cristae structure (66). On the other hand, treating BMSCs with dexamethasone downregulates DRP1, MFF, and FIS1 (34). Thus, further research is needed to clarify how GCs alter mitochondrial dynamics. Nevertheless, studies have identified several molecules that alter the dynamics in ways that may alleviate SONFH. Blockade of DRP1 helps protect osteoblasts from oxidative stress by inhibiting excessive mitochondrial fission (67). Amyloid precursor protein, which is expressed in bone cells and many other cell types (68), stimulates mitochondrial fusion to promote osteoblast survival and bone formation in the presence of oxidative stress. At the same time, the protein may promote antioxidant responses to ROS and prevent cytochrome C release, helping prevent osteoblast apoptosis (69).

## Mitochondrial ROS metabolism

Mitochondria are at once the centers of ROS production in cells and the organelles most sensitive to ROS (21). There are two antioxidant systems according to the distribution of antioxidant enzymes in the cell, and the enzymes in mitochondria and cytoplasm can both significantly clear the excessive ROS in the cell. Nuclear factor erythroid 2-related factor 2 (Nrf2) is a vital transcription factor that is dysregulated in various oxidative stress-related pathologies (70). It combats intracellular oxidative stress by activating multiple downstream genes, such as Heme Oxygenase-1 (HO-1), superoxide dismutase (SOD), glutathione (GSH), and catalase (CAT) (71, 72), by binding to the upstream antioxidant response element (ARE) sequence (73). The phosphatase and tensin homolog inhibitor VO-OHpic attenuates GC-associated endothelial progenitor cell dysfunction and osteonecrosis of the femoral head by activating Nrf2 signaling and inhibiting the mitochondrial apoptosis pathway (66). PARK7 promotes repair in early SONFH by enhancing resistance to stress-induced apoptosis in BMSCs via regulation of the Nrf2 signaling pathway (74). In addition, mitochondria contain a strong antioxidant system involving SOD2, Gpx1/4, Prx3, Trx2, and TrxR2 (75). SOD2, for example, can eliminate excess superoxide and protein oxidation in mitochondria during osteoblast differentiation. To promote this activity, the enzyme is deacetylated at lysine 68 by SIRT3 (35). Melatonin can target mitochondria to promote the activity of SIRT3 and SOD2 to reduce oxidative stress, which has been shown to promote osteoblast survival and increase bone mass (76, 77). These considerations illustrate the possibility of treating SONFH by increasing the antioxidant capacity of mitochondria or their host cells.

**Figure 4:**
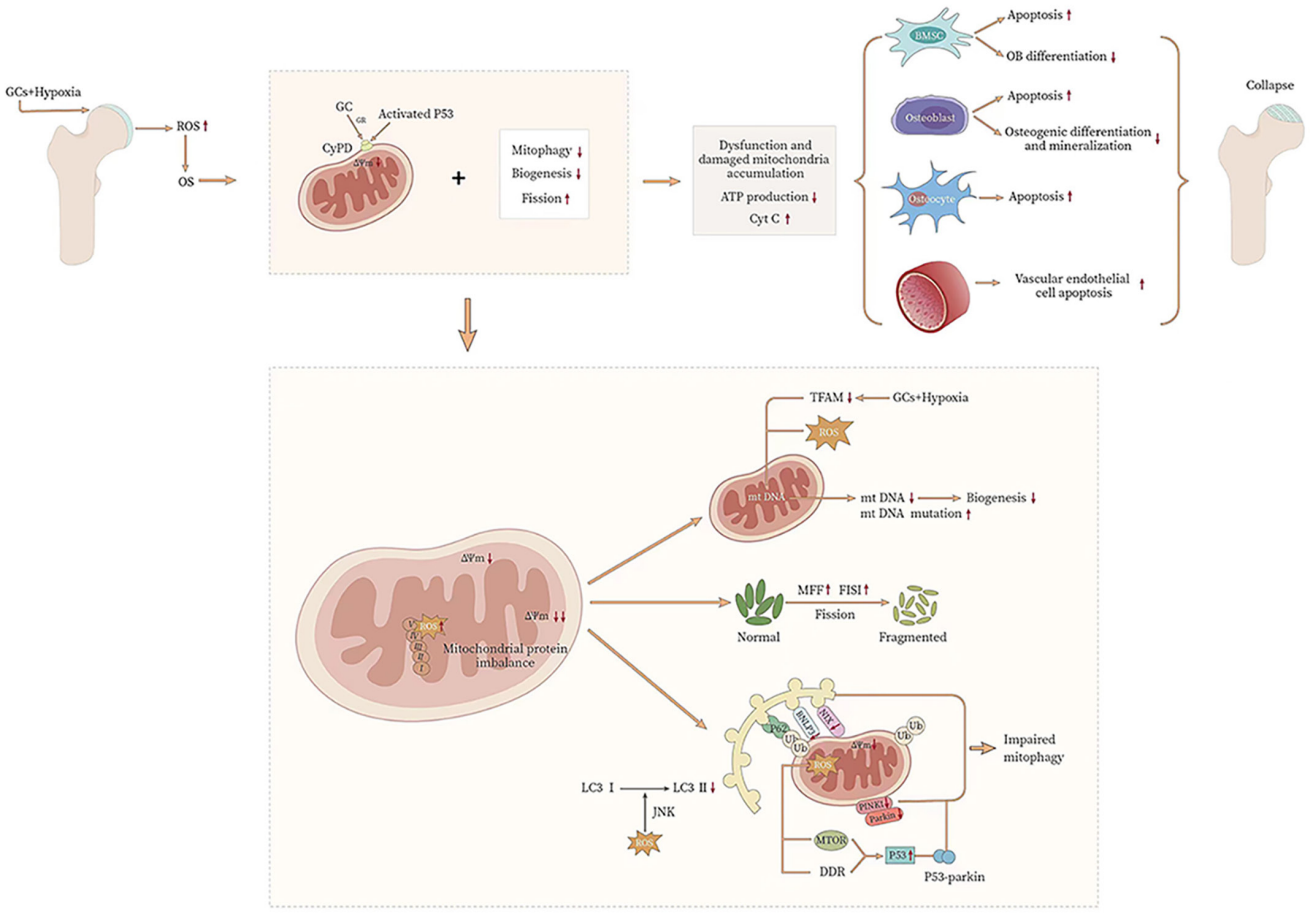
The regulatory mechanisms of mitochondrial homeostasis in SONFH. Under the combined influence of GCs and ischemia-hypoxia on the femoral head, local levels of ROS increase, leading to heightened oxidative stress. The suppression of mitophagy and mitochondrial biogenesis, excessive fission, and disorder of mitochondrial ROS metabolism lead to the dysfunction of mitochondrial homeostasis. These changes impair mitochondrial function, reduce ATP production, and lead to the release of cytochrome C into the cytosol. As a result, apoptosis rates in BMSCs, osteoblasts, osteocytes, and vascular endothelial cells increase, accompanied by reduced differentiation and mineralization of osteoblasts. These effects collectively contribute to the collapse of the femoral head. GCs are associated with ischemic and hypoxic states, as well as excessive ROS inhibiting TFAM, leading to decreased mitochondrial gene expression, increased mutations, and decreased mitochondrial biological responses. Meanwhile, elevated expression of genes such as MFF and FIS1 leads to increased mitochondrial division and functional impairment. Dysregulation of mitophagy pathways, including decreased PINK1, Parkin, and LC3-II expression, impairs mitophagy.

Figure 4 shows and depicts the relevant regulatory mechanisms of mitochondrial homeostasis in SONFH.

## Conclusions

Accumulating evidence suggests that the dysfunction of mitochondrial homeostasis helps drive SONFH. The suppression of mitophagy and mitochondrial biogenesis, excessive fission, and disorder of mitochondrial ROS metabolism lead to the dysfunction of mitochondrial homeostasis.

Efforts to target the major components of that homeostasis-mitochondrial biogenesis, mitophagy, mitochondrial dynamics, and mitochondrial antioxidant capacity – may be effective against this debilitating condition. Future research should focus on regulating these processes to restore mitochondrial homeostasis. Potential therapeutic targets include enhancing mitophagy via PINK1/Parkin, promoting mitochondrial biogenesis through TFAM and PGC-1α, balancing mitochondrial dynamics by inhibiting DRP1, and reducing oxidative stress with antioxidants like melatonin and SIRT3 activators. Exploring natural compounds, such as those in traditional Chinese medicine, may also offer novel treatments. However, it should be noted that mitochondrial activity is ubiquitous across all tissues and involved in numerous physiological processes. Therefore, we expect new drug interventions targeting mitochondria to be able to selectively affect the femoral head to minimize systemic side effects. Continued investigation into these pathways and their clinical applications is essential for developing effective therapies for SONFH.

## ICMJE Conflict of Interest Statement

The authors declare that the study was conducted in the absence of any commercial or financial relationships that could be construed as a potential conflict of interest.

## Funding Statement

This work is supported in China by the National Natural Science Foundationhttp://dx.doi.org/10.13039/501100001809 (82074472) and Zhejiang Provincial Natural Science Foundation (LQ22H060003).

## Acknowledgement

We would like to thank A Chapin Rodríguez PhD for English language editing.
